# Xeroderma Pigmentosum – A case report with oral implications

**DOI:** 10.4317/jced.50727

**Published:** 2012-10-01

**Authors:** Camila Lopes-Cardoso, Luciana M. Paes da Silva Ramos Fernandes, Julierme Ferreira-Rocha, Cleverson Teixeira-Soares, Jaison Antônio-Barreto, José Humberto-Damante

**Affiliations:** 1 DDS, MSc, PhD student, Department of Stomatology, Bauru School of Dentistry, University of São Paulo, Bauru, São Paulo, Brazil.; 2 DDS, MSc. Department of Stomatology, Bauru School of Dentistry, University of São Paulo, Bauru, São Paulo, Brazil.; 3 MD, PhD. Lauro de Souza Lima Institute, Bauru, São Paulo, Brazil.; 4 DDS, MSc, PhD. Department of Stomatology, Bauru School of Dentistry, University of São Paulo, Bauru, São Paulo, Brazil.

## Abstract

Xeroderma Pigmentosum is a rare autosomal recessive genetic disorder characterized by defective DNA repair leading to clinical and cellular hypersensitivity to ultraviolet radiation and carcinogenic agents. Important clinical features are: intense cutaneous photosensitivity, xerosis, poikiloderma, actinic keratosis, acute burning under minimal sun exposure, erythemas, hyperpigmented lentiginous macules, and malignant lesions in sun-exposed areas, including basocellular carcinoma, squamous cell carcinoma, and melanoma. There is a great involvement of many parts of the body, especially head and neck. The oral manifestations are mainly related to the occurrence of malignant tumors in the lips, tongue and buccal mucosa. This paper reports a rare case of Xeroderma Pigmentosum in a 41-year-old male presenting mainly dermatological, neurological and ophthalmological involvement. Oral implications such as severe oral pain and mouth opening limitation were present due to perioral scars. In addition, this paper discuss some important aspects concerning the role of the dental professional management of this entity, since XP patients require constant dental care and follow-up in order to control the occurrence of new lesions on the lips or inside oral cavity.

** Key words:**Actinic cheilitis, oral involvement, Xeroderma pigmentosum.

## Introduction

Firstly described by Hebra and Kaposi in 1874, Xeroderma Pigmentosum (XP) is a rare autosomal recessive genetic disorder characterized by defective DNA repair which leads to clinical and cellular hypersensitivity to ultraviolet radiation and other carcinogenic agents (1-6). Important clinical features are: intense cutaneous photosensitivity, xerosis, poikiloderma, actinic keratosis, acute burning under minimal sun exposure, erythemas, hyperpigmented lentiginous macules, and malignant lesions in sun-exposed areas, including basocellular carcinoma, squamous cell carcinoma, and melanoma. The epidemiology rates of this disorder are varied. Authors described different prevalence rates such as: 1:20,000 in Japan, 1:250,000 in the USA and approximately 2.3 per million in Western Europe (1-9). Eight forms of the disease are recognizable according to the features of the molecular defect and clinical aspects (4). High frequency of consanguinity has been reported. Approximately 80% of XP have ocular complications, and neurological affection occur in approximately 20% of cases (1-6). The face and particularly the lips are highly exposed to UV radiation and other carcinogenic agents. The aim of the present paper is to report a XP case with oral implications and to discuss the role of the dental professional management of this entity.

## Case Report

**Figure 1:**
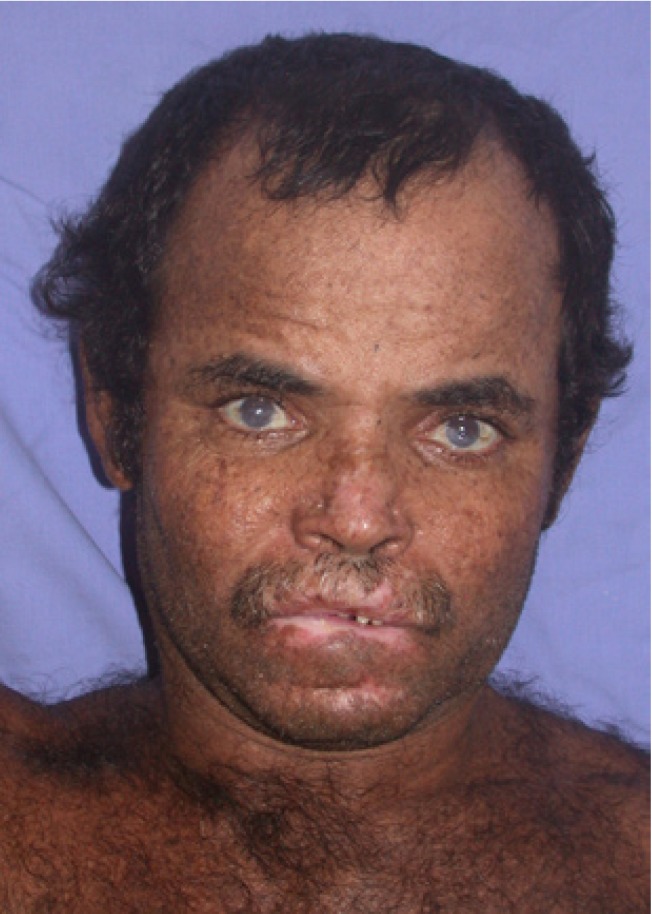
Hyperpigmented macules and papules in the skin face, perioral scars and ophthalmic involvement.

**Figure 2:**
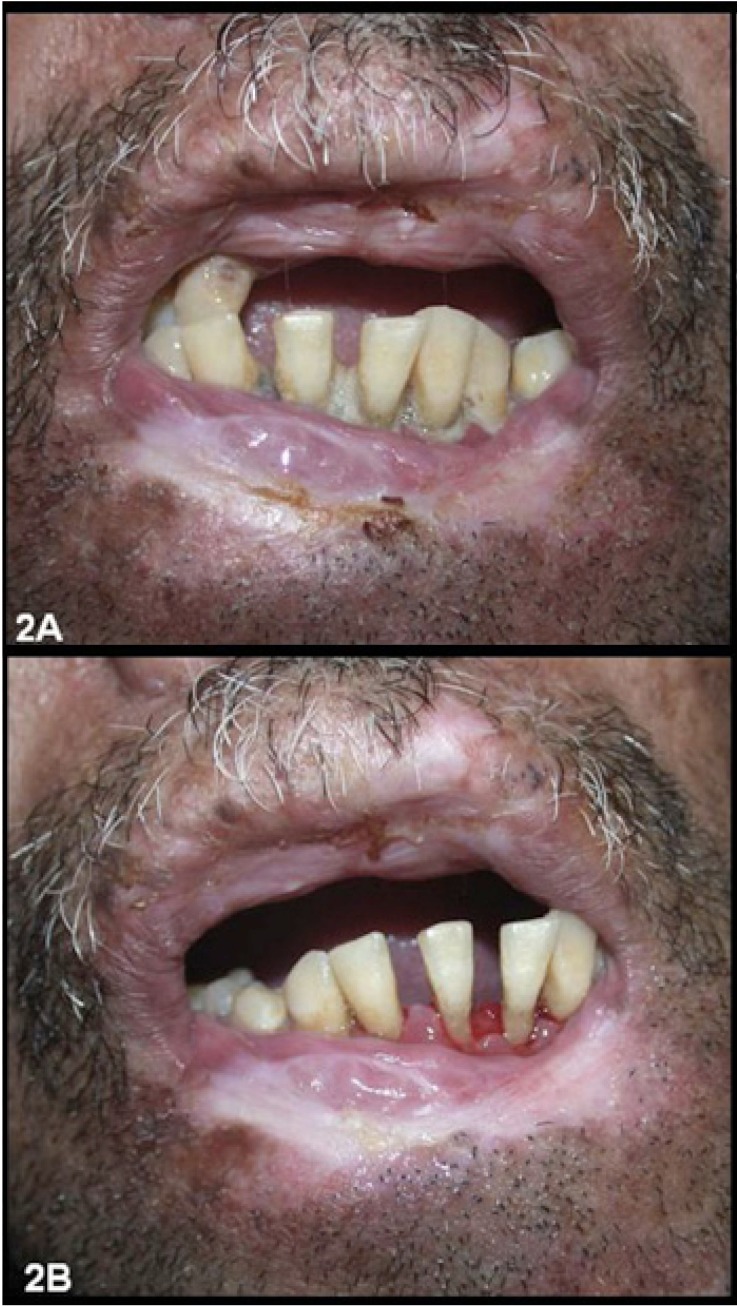
2A. Perioral scars, dental plaque due to poor oral hygiene and microstomy. 2B. Oral condition after dental plaque removal using ultrasound.

**Figure 3:**
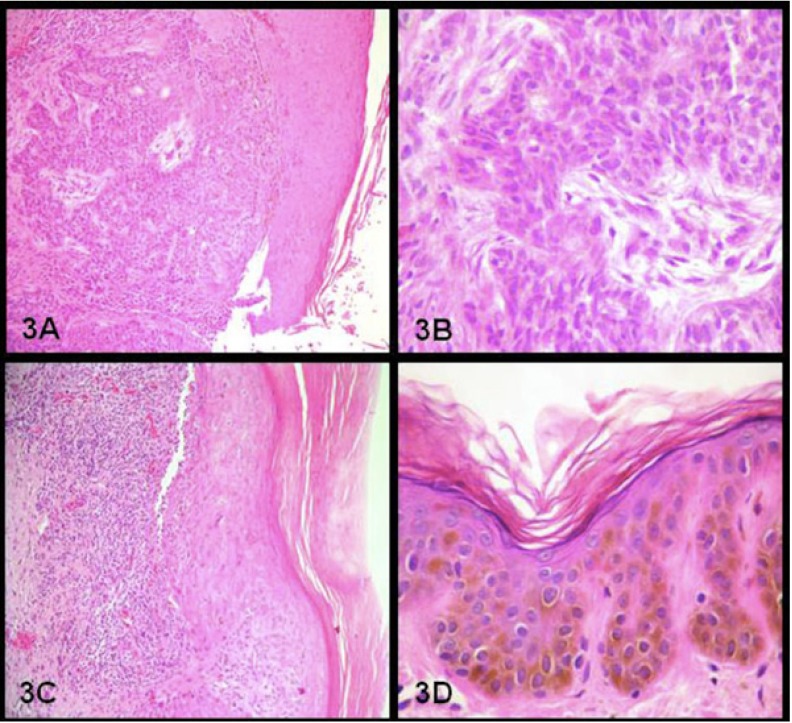
3A,3B- Basal cell carcinoma in facial skin: Blocks of atypical basaloid cells infiltrating the stroma (40x, 200x HE);3C-Actinic keratosis lichenoides: epithelial hyperplasia and dysplasia associated with infiltrate lichenoid in the dermis (HE 200x); 3D-Lentigo simplex in skin: basal cell layer hyperpigmentation associated with an intense loss of melanin pigment in the dermis (400x HE).

A 41-year-old male sought our Service of Stomatology complaining about severe oral pain and mouth opening limitation due to perioral scars. He presented hyperpigmented macules and papules all over the skin (Fig. 1), short stature, difficulty in speaking, and tremors during writing and moving. Oral hygiene was extremely poor due to microstomy (Fig. 2). During medical history assessment, he reported the removal of three skin lesions nine years before; two of them of facial skin (basal cell carcinoma and actinic keratosis lichenoides) and another one at the chest skin (lentigo simplex) (Fig. 3). The patient had consanguineous parents and a brother with the same disease. Dental and periodontal problems were observed during clinical examination and in panoramic radiograph. An ultrasonic cleaning was performed and limitation to reach the teeth occurred due to microstomy (Fig. 2). He was instructed to perform proper hygiene of the mouth as well as to keep a rigorous photoprotection on the lips and the rest of the body by using sunscreen moisturizers, wide-brimmed hat and appropriate clothing. Furthermore, the patient was referred to dermatological, neurological and ophthalmological treatment in order to have a multidisciplinary management of the case.

## Discussion

Skin changes are typical features of XP, such as persistent erythema of the skin. In many cases, these symptoms may appear immediately after birth or within the following three years, but it may not develop until late childhood or may not be recognized until adulthood (1-6). Other features of XP are discolorations, weakness and fragility, skin scarring, neurological and ocular disorders (10-11). The differential diagnosis of XP should be performed with two other syndromes caused by mutations of excision repair pathway genes: Cockayne’s syn-drome (CS) and Trichothiodystrophy (TTD). However, the occurence of skin cancer associated to high skin sensitivity to UV radiation is commonly observed in XP, but not in CS or TTD cases (12). The final diagnosis of XP can be confirmed by special laboratory tests by examining the DNA damage repair in cells from cultures exposed to ultraviolet radiation. The most common tests are skin biopsy and culture of skin fibroblasts.

De Sanctis Cacchione syndrome has been associated in very few patients XP groups A and D, which represents the most aggressive disease (13). In the past, any association of XP and neurological disorders was considered as De Sanctis-Cacchione syndrome. Currently, the diagnostic criteria include severe neurological disease such as microcephaly with progressive mental deterioration, hyporeflexia or areflexia, choreoathetosis, ataxia, spasticity, shortening of Achilles tendon with eventual quadriparesis, markedly retarded growth and immature sexual development. In addition, patients can present epilepsy, progressive sensorineural deafness and abnormal electroencephalographic findings (14). In our case, although the patient had some neurological symptoms, the intensity and the signs described above were not enough to classify this association.

The oral manifestations are mainly related to the occurrence of malignant tumors in the lips, tongue and buccal mucosa (11). In our case, microstomy was a result of successive labial plasty for the treatment of actinic cheilitis. Actinic cheilitis is a potentially malignant lesion that affects the lower lip of white patients who were frequently exposed to sun. Pain is a consequence of fibrous area that stretches when the patients opens the mouth for feeding, speaking, breathing, and for oral hygiene performance. Therefore, the patient has poor hygiene habits and consequently, a high rate of dental plaque, caries and periodontal disease.

There is no cure for XP. Treatment consists of minimizing exposure to sunlight and regular dermatological care, as well as surgery for recurrent tumors excision. Early diagnosis and extensive sun protection have the potential to prevent skin cancers in XP patients and prolong their life expectancy. Although the management of XP is complicated due to the difficulty in avoiding daily UV exposure, sun protection can be achieved by wearing protective clothing, UV-absorbing sunglasses with side shields and the use of topic sunblocking agents. UV-absorbing films and filters can be placed over windows and fluorescent or halogen lamps. Self- and dermatological skin examinations to monitor any skin changes and ensure early detection and treatment of skin cancers should be regularly performed. Also, an ophthalmologist must be involved since the beginning of the management of these patients (6). Eye care consists of sunglasses, artificial tears, steroid drops, and bland ointment at night. These are essential components of a prevention program. Patients should be followed up every 3 months (1).

Genetic counseling is also an important component of XP patient management, especially in a family that has an affected child and is considering having more children. XP patients have 1,000 times more chances of developing early skin cancer including squamous cell carcinoma, basal cell carcinoma and melanoma, less frequently (1-6).

Dental approach is important for the management of XP patients with cancer in oral and perioral region. Clinical examination carried out regularly by the dentist is mandatory for the detection of premalignant or malignant lesions. Furthermore, it is necessary to establish protocols for prophylaxis and topical fluoride application, as well as the use of chlorhexidine digluconate 0.12%, aiming the homeostasis of the oral environment. The use of mouthwashes with high alcohol concentration should be avoided because there is an increased risk of developing oral cancer in these patients (15). Furthermore, regular dental procedures, such as dental extraction, restoration and rehabilitation become a challenge for the dentist due to difficult access to the oral cavity. The XP patient needs a special dental care and a conventional treatment plan is not always possible to perform. In our case, the ultrasonic cleaning was difficult to perform due to microstomy. In some situations, sedation or general anesthesia should be considered for regular procedures in Dentistry.

Finally, the main oral manifestations of XP are actinic cheilitis, basal cell and squamous cell carcinomas. In addition, the presence of scars and lip repair surgery sequels are commonly observed, resulting in difficult oral care and hygiene. Besides dermatological, ophthalmological and neurological management, XP patients require constant dental care and follow-up in order to control the occurrence of new lesions on the lips or inside oral cavity.
